# Microglial Pdcd4 deficiency mitigates neuroinflammation-associated depression via facilitating Daxx mediated PPARγ/IL-10 signaling

**DOI:** 10.1186/s12974-024-03142-3

**Published:** 2024-05-31

**Authors:** Yuan Li, Bing Zhan, Xiao Zhuang, Ming Zhao, Xiaotong Chen, Qun Wang, Qiji Liu, Lining Zhang

**Affiliations:** 1 Key Laboratory of Infection and Immunity, Department of Immunology, School of Basic Medical Sciences, Cheeloo College of Medicine, Shandong University, 44# Wenhua Xi Road, Jinan, 250012 Shandong China; 2 Key Laboratory of Endocrine Glucose & Lipids Metabolism and Brain Aging, Department of Endocrinology, Shandong Provincial Hospital Affiliated to Shandong First Medical University, Jinan, Shandong China; 3 Key Laboratory for Experimental Teratology of the Ministry of Education, Department of Medical Genetics, School of Basic Medical Sciences, Cheeloo College of Medicine, Shandong University, Jinan, Shandong China

## Abstract

The dysregulation of pro- and anti-inflammatory processes in the brain has been linked to the pathogenesis of major depressive disorder (MDD), although the precise mechanisms remain unclear. In this study, we discovered that microglial conditional knockout of Pdcd4 conferred protection against LPS-induced hyperactivation of microglia and depressive-like behavior in mice. Mechanically, microglial Pdcd4 plays a role in promoting neuroinflammatory responses triggered by LPS by inhibiting Daxx-mediated PPARγ nucleus translocation, leading to the suppression of anti-inflammatory cytokine IL-10 expression. Finally, the antidepressant effect of microglial Pdcd4 knockout under LPS-challenged conditions was abolished by intracerebroventricular injection of the IL-10 neutralizing antibody IL-10Rα. Our study elucidates the distinct involvement of microglial Pdcd4 in neuroinflammation, suggesting its potential as a therapeutic target for neuroinflammation-related depression.

**Supplementary Information:**

The online version contains supplementary material available at 10.1186/s12974-024-03142-3.

## Introduction

The neuroinflammatory process has been associated with a range of psychiatric disorders, including schizophrenia, depression, and anxiety [1]. Accumulating shreds of evidence support the relationship between inflammatory cytokines and affective disorders, including interleukin (IL)-1β, IL-6, and tumor necrosis factor-α (TNF-α) [2]. These results have been confirmed by rodent models, in which mental stress, in addition to leading to inflammatory immune activation, produces depressive-like behavior [3]. Although studies have focused on the effect of pro-inflammatory factors on depression, the role of anti-inflammatory cytokines and the mechanisms underlying the imbalance between pro- and anti-inflammatory factors need to be explored.

Inflammatory stimuli are sensitive to microglia, a resident macrophage in the brain, and it has an immune response function. Under stress conditions, microglia transform into an active state and participate in emotional behaviors through activity-dependent regulation of synaptic pruning [4, 5]. Peripheral lipopolysaccharide (LPS), an agent for microglial activation, induces microglial pro-inflammatory reactions and depressive-like behavior in mice [6]. Otherwise, the long-term treatment of minocycline, a microglial inhibitor, prevented mice from experiencing depressive-like behavior due to neuroinflammation [7, 8]. Peroxisome proliferator-activated receptor gamma (PPARγ) belongs to the nuclear receptor family, which is a ligand-dependent transcription factor. The dysfunction of PPARγ is accompanied by mental illness, which is a genetic risk factor for depression [9, 10]. Based on the fact that PPARγ play a key role in depression. Such as, neuronal PPARγ knockout in the prefrontal cortex of mice induces depressive-like behavior. Conversely, the PPARγ agonist, Rosiglitazone, has an antidepressant-like effect on stressed mice [10–12]. One of the reasons is that PPARγ signaling promotes neuroplasticity and neurogenesis by boosting brain-derived neurotrophic factor (BDNF) [13]. Additionally, PPARγ activation results in inhibition of inflammatory responses by increasing anti-inflammatory cytokines expression or suppressing pro-inflammatory molecules, which helps damaged brain repair [14, 15]. However, the microglia specific mechanism of PPARγ in emotional behavior is still unclear.

Programmed cell death 4 (Pdcd4) an apoptosis-related molecule, extensively participates in tumorigenesis and inflammatory diseases. Our previous reports have demonstrated Pdcd4 is elevated in brain of patient with depression, and neuronal-expressed Pdcd4 is involved in stress-induced depressive-like behaviors, by blocking BDNF and up-regulating proinflammatory response [16, 17]. Pdcd4 has a neuroinflammatory effect on emotional disorders, however, its biological function in central nervous system (CNS) neuroinflammation remains unclear. Here, we demonstrated that microglial PPARγ nuclear translocation involves inflammatory-related depression. LPS led to the upsurge in Pdcd4 expression in the microglias of depressed mice, and microglial Pdcd4 conditional knockout had antidepressant effects on mice. Functionally, overexpressed Pdcd4 promotes microglia activation by decreasing the levels of IL-10. Mechanically, we discovered that Pdcd4 competed with Daxx to block PPARγ nuclear translocation, resulting in interruption of IL-10 transcription. In this study, we emphasized that cellular PPARγ distribution regulates neuroinflammation, and microglial Pdcd4 plays a critical role in that.

## Results

### Microglial knockout of Pdcd4 ameliorates neuroinflammation-related depression

**Fig. 1:**
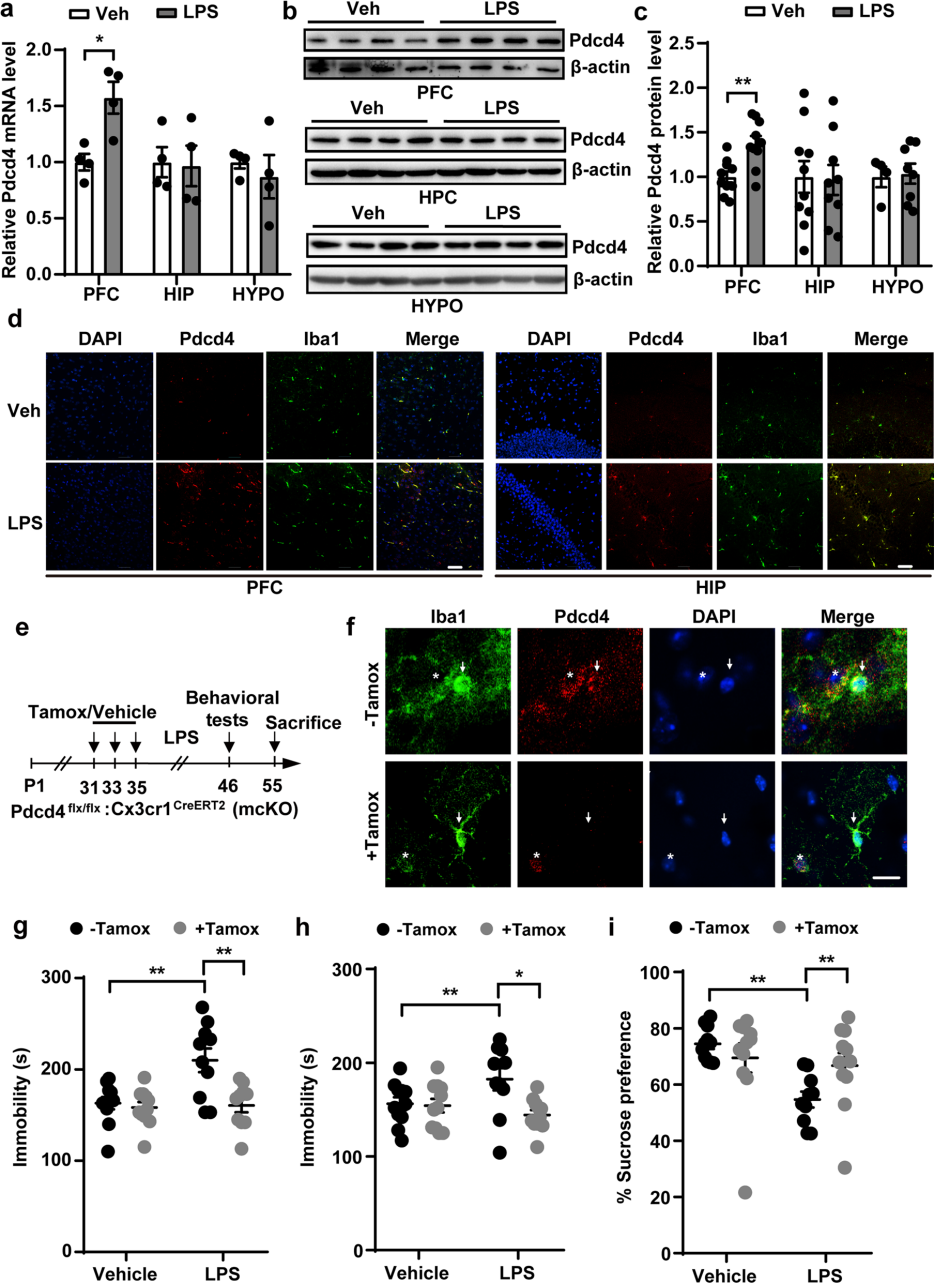
Microglial knockout of Pdcd4 ameliorates neuroinflammation related depression. **a** The change of mRNA levels of Pdcd4 in the prefrontal cortex (PFC), the hippocampal (HIP) and the hypothalamus (HYPO) after LPS administration for 10 days. Unpaired two-tailed Student’s t test, *P < 0.05. **b**, **c** The change of protein levels of Pdcd4 in the prefrontal cortex (PFC), the hippocampal (HIP) and the hypothalamus (HYPO) after LPS administration for 10 days. Unpaired two-tailed Student’s t test, **P < 0.01. **d** Iba1 antibody (Green), Pdcd4 antibody (Red) and DAPI (blue) was used into immunostaining in the PFC or HIP. N = 3 per group, scale bar = 50 μm. **e** Schematic diagram of animal experiments of LPS-induced mice. **f** Iba1 (Green) and Pdcd4 (Red) immunostaining in the PFC. Arrow represents microglia, star represents cell which is adjacent to and surrounded by microglia. N = 3 per group, scale bar = 20 μm. **g** Immobility time in TST (Veh vs. LPS: F_1,39_ = 8.259, P < 0.01; − Tam vs. + Tam: F_1,39_ = 9.949, P < 0.01), **h** immobility time in FST (Veh vs. LPS: F_1,39_ = 1.025, P = 0.317; − Tam vs. + Tam: F_1,39_ = 6.401, P < 0.01), and **i** sucrose consumption in SPT under basal or LPS conditions in the mcKO mice with or without Tamoxifen (Veh vs. LPS F_1,39_ = 8.506, P < 0.01; − Tam vs. + Tam F_1,39_ = 0.846, P = 0.363). Two-ways ANOVA and Sidak’s multiple comparisons test, *P < 0.05, **P < 0.01

To explore intermediate components that modulate neuroinflammation-related depression, we exposed adult male mice to LPS intraperitoneally for 10 days to mimic depression. (Fig. S1a). Validation of mice with depressive-like phenotypes was achieved by the use of tail suspension test (TST) and forced swimming test (FST) (Fig. S1b, c). We found that Pdcd4 mRNA and protein levels are increased in the prefrontal cortex (PFC) but not in the hippocampus (HIP) of the LPS-induced depressive mice (Fig. 1a–c). Microglias are the response of the cell to LPS triggering neuroinflammation in the central nervous system. To explore whether the expression of Pdcd4 in microglia is elevated after LPS injection, we performed immunofluorescence staining experiments on mouse brain slices using Pdcd4 antibody and Iba1 antibody. The results showed that LPS treatment augmented the level of Pdcd4 in microglias of the PFC and the HIP (Fig. 1d). To investigate the specific effect of microglial Pdcd4 on LPS-induced depressive-like behaviors, we generated Pdcd4 mcKO mice by crossing the floxed Pdcd4 allele (Flx) mice with the Cx3cr1-CreERT2 mice (Fig. 1e). Firstly, we isolated the microglial cells in mcKO with tamoxifen and the control without tamoxifen mice by flow cytometry. Western blot showed that Pdcd4 was deleted in microglia in the mcKO mice with tamoxifen (Fig. S2a, b). Next, immunofluorescence histochemistry of brain slices showed Pdcd4 expression diminished in the Iba1+ cells of Pdcd4 mcKO mice-treated with tamoxifen (Fig. 1f). We then assessed the depressive-like behaviors in microglial Pdcd4 knockout mice exposed to LPS by using FST, TST, and sucrose preference test (SPT). As expected, LPS led to depressive-like behaviors in the mcKO mice without tamoxifen, as indicated by increased immobility and decreased percentage of sucrose preference, which were reversed by the conditional knockout of Pdcd4 in microglia, without affecting the locomotor activity (Fig. 1g–i; Fig. S2c). Interestingly, the LPS-induced increase in anxiety-like behaviors in control mice was not reversed by microglial Pdcd4 knockout (Fig. S2d, e). These results indicate that Pdcd4 deficiency in microglia protects mice from neuroinflammation-related depressive-like phenotype.

### Microglial Pdcd4 deficiency mitigates LPS-induced microglial activation via facilitating the PPARγ signaling

**Fig. 2:**
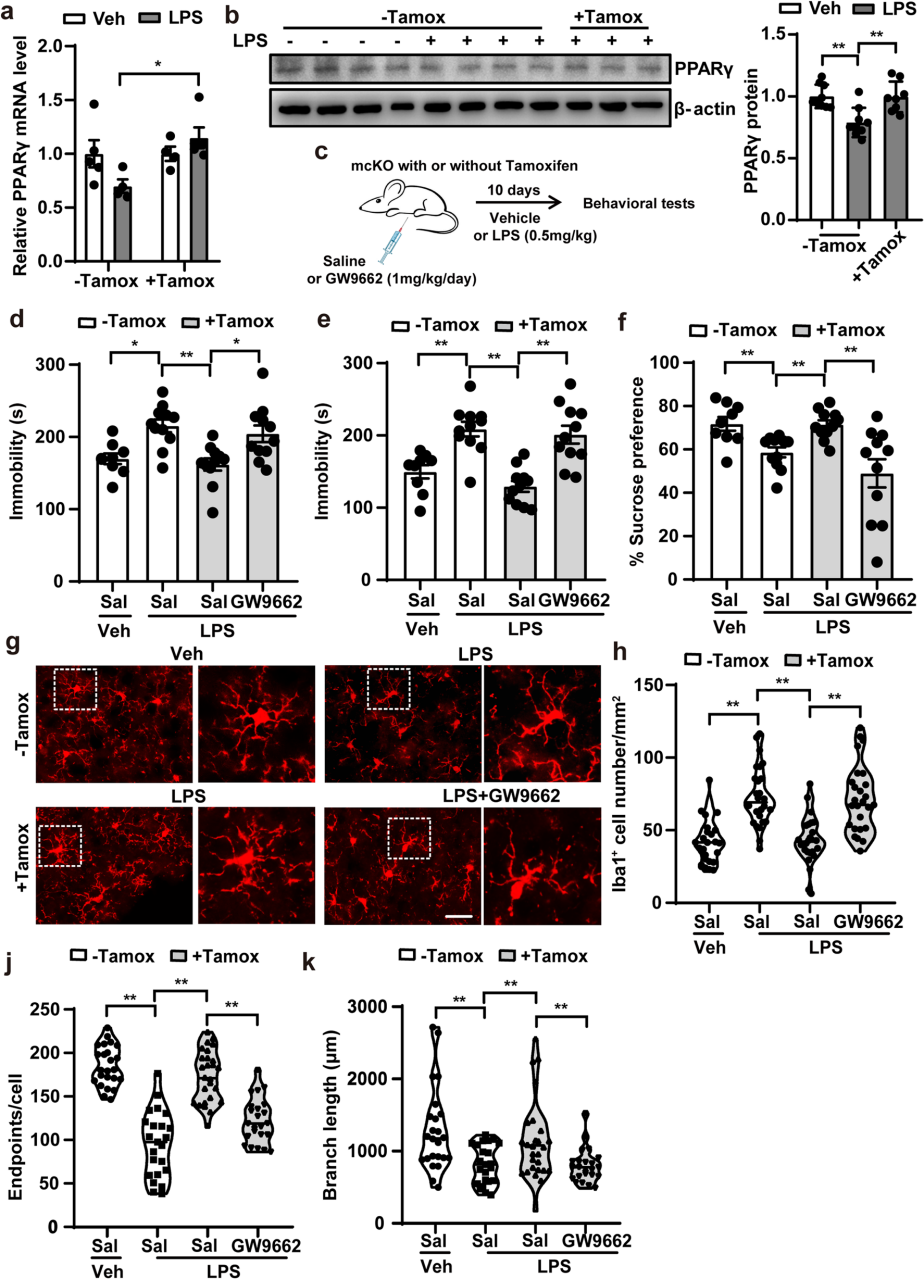
Microglial Pdcd4 deficiency mitigates LPS-induced microglial activation via facilitating the PPARγ signaling. **a** The change of mRNA levels of PPARγ in the PFC of mcKO mice with or without tamoxifen after LPS administration for 10 days. Two-ways ANOVA and Sidak’s multiple comparisons test (Veh vs. LPS F_1,14_ = 0.6, P = 0.45; − Tam vs. + Tam F_1,14_ = 5.168, P < 0.05), *P < 0.05. **b**, **c** The change of protein levels of PPARγ in the PFC of control or tamoxifen treatment mcKO mice after LPS administration for 10 days. One-way ANOVA and Tukey’s multiple comparisons test (F_2,23_ = 10.15, P < 0.01), **P < 0.01. **c** Time course of GW9662/LPS administration and behavior tests. Mice injected GW9662 for 30 min before LPS challenge. **d** Immobility time in TST (F_3,38_ = 7.761, P < 0.01), **e** immobility time in FST (F_3,38_ = 15.09, P < 0.01), and **f** sucrose consumption in SPT (F_3,38_ = 7.531, P < 0.01). One-way ANOVA and Tukey’s multiple comparisons test, *P < 0.05, **P < 0.01. **g**, **h** Iba1 staining density in the PFC. N = 3 per group, One-way ANOVA and Tukey’s multiple comparisons test, **P < 0.01, scale bar 100 μm. **i**–**k** Characteristic morphological changes of microglia in the PFC by Iba1 staining. n = 3 per group, one-way ANOVA and Tukey’s multiple comparisons test (cell number F_3,163_ = 34.99, P < 0.01; endpoints F_3,98_ = 51.42, P < 0.01; branch F_3,98_ = 7.39, P < 0.01), **P < 0.01

To explore the molecular mechanisms underlying the antidepressant effect observed in the microglial Pdcd4 deletion, we analyzed the mRNA expression profiles in the PFC of LPS-treated microglial Pdcd4 knockout by RNA sequencing (RNA-seq). 586 genes were differentially expressed in the PFC of the Pdcd4 microglia conditional knockout mice in response to LPS compared to the LPS-treated control (Fig. S3a). KEGG pathway analysis showed thermogenesis and oxidative phosphorylation were significantly changed. Gene Set Enrichment Analysis (GSEA) indicated the PPAR signaling pathway was enriched in the LPS-treated Pdcd4 microglial knockout group, among which PPARγ might be the core gene that was regulated (Fig. S3b–d). To clarify whether PPARγ is a core gene in the mice resistant to LPS-induced depressive-like behavior, we first measured the mRNA and protein levels of PPARγ in the PFC in response to LPS. Compared to the vehicle-treated group, PPARγ was markedly decreased in the PFC of the LPS-treated control mice but not the mcKO mice with tamoxifen (Fig. 2a, b). GW9662, a PPARγ inhibitor, was able to eliminate the antidepressant effect in microglial Pdcd4 deletion in mice by injecting it into mice simultaneously following LPS administration (Fig. 2c–f), but it did not exacerbate depressive-like behavior in WT mice under LPS conditions (Fig. S4a, b). Together, these data suggest that the antidepressant effect of microglial Pdcd4 deficiency in response to LPS might be mediated by the PPARγ signaling.

Next, we investigated the activation of microglia in the PFC of mice. The number of microglial cells in the PFC of the mcKO mice without tamoxifen was significantly increased after LPS treatment. However, when we treated the mice simultaneously with LPS and GW9662, the number of microglial cells was also obviously up-regulated compared to the LPS-treated mcKO with tamoxifen group (Fig. 2g, h). Similarly, the number and length of microglial cell branches were reduced after LPS injection in control mice but not in the Pdcd4 microglia conditional knockout mice. Inhibition of the PPARγ signaling with GW9662 also decreased the number and length of microglial cell branches in the mcKO with tamoxifen mice in response to LPS (Fig. 2i–k). These results suggest that microglial Pdcd4 deficiency mitigates LPS-induced microglial activation via facilitating the PPARγ signaling pathway.

### Pdcd4 regulates the nuclear and cytoplasmic translocation of PPARγ in an LPS-independent manner

**Fig. 3:**
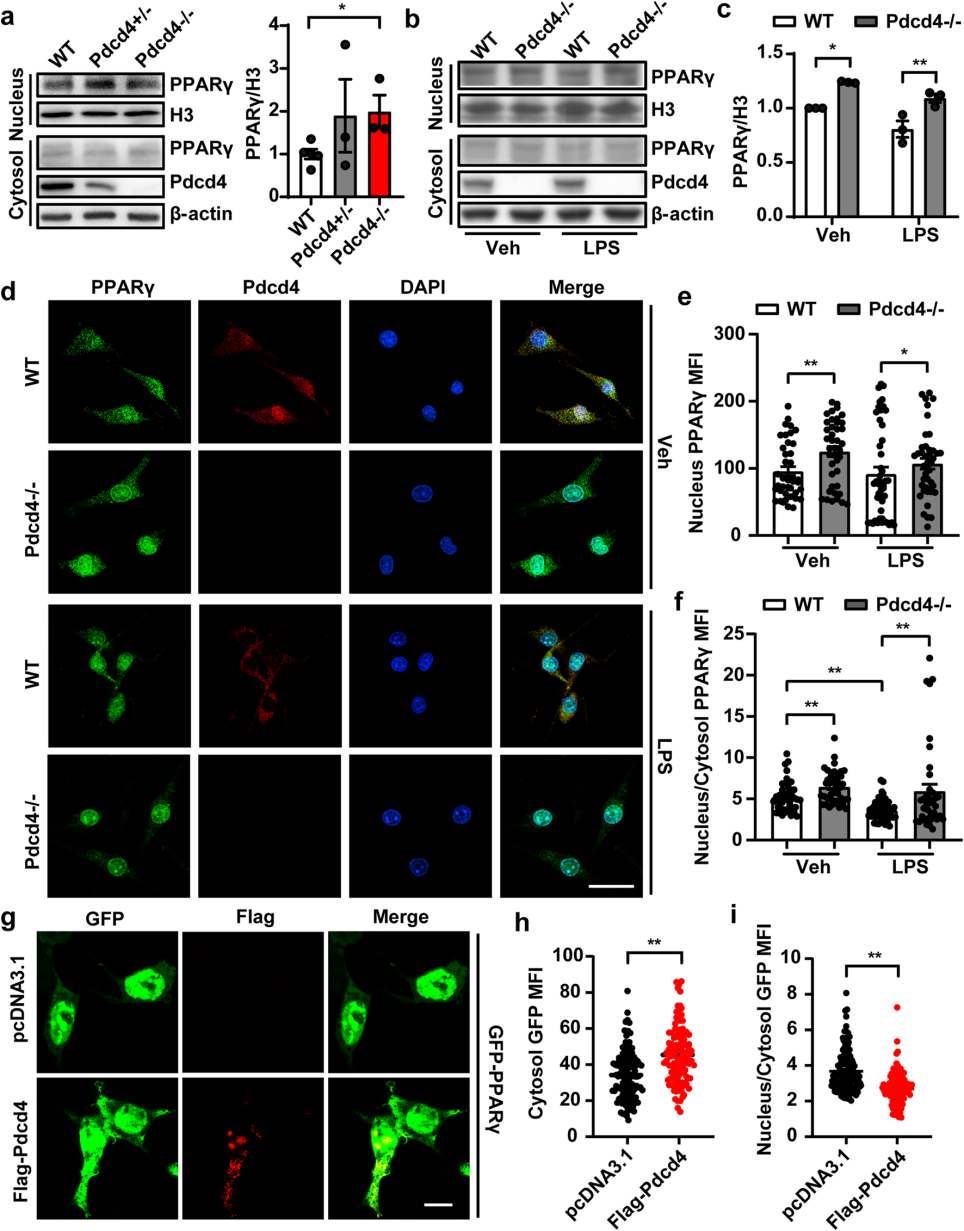
Pdcd4 regulates the nuclear and cytoplasmic translocation of PPARγ in an LPS-independent manner. **a** Nuclear and cytoplasmic proteins were isolated from the PFC of Pdcd4 gene knockout heterozygous (Pdcd4+/−), Pdcd4 gene knockout homozygous (Pdcd4−/−) and littermate control mice, and determined by western blot. n = 3–4 per group, one-way ANOVA and Tukey’s multiple comparisons test (F_2,8_ = 1.765, P = 0.23), *P < 0.05. **b** Nuclear and cytoplasmic proteins were isolated from the primary microglia of Pdcd4−/− and littermate control mice with or without 1 µg/mL LPS treatment for 24 h, and determined by western blot. **c** Relative grey intensity analysis of nucleus PPARγ and H3, n = 3 per group, two-ways ANOVA and Sidak’s multiple comparisons test (Veh vs. LPS F_1,8_ = 15.85, P < 0.01; WT vs. Pdcd4−/− F_1,8_ = 37.73 P < 0.01), *P < 0.05, **P < 0.01. **d** Subcellular localizations of PPARγ were observed by immunofluorescence analysis with PPARγ staining in WT and Pdcd4−/− primary microglias. **e**, **f** The mean fluorescence intensity (MFI) of PPARγ was quantified from n > 30 cells; scale bar 20 μm. 1 µg/mL LPS treamtment for 24 h, two-ways ANOVA and Sidak’s multiple comparisons test (Nuclear MFI: Veh vs. LPS F_1,166_ = 1.746, P = 0.188; WT vs. Pdcd4−/− F_1,166_ = 7.166, P < 0.01; Ratio MFI: Veh vs. LPS F_1,166_ = 5.13, P < 0.05; WT vs. Pdcd4−/− F_1,166_ = 14.65 P < 0.01), *P < 0.05, **P < 0.01. **g**–**i** HEK293 cells were transfected into GFP-tagged full-length PPARγ by accompany with pcDNA3.1 or Flag-Pdcd4 plasmid for 24 h, and localizations of GFP were observed by immunofluorescence, and the mean fluorescence intensity (MFI) of GFP was quantified from n > 30 cells; scale bar 20 μm. Unpaired two-tailed Student’s t test, **P < 0.01. All independent experiments were repeated for three times

To ensure whether or how Pdcd4 regulates the function of PPARγ, mouse primary microglias were taken into the experiment. Purified mouse primary microglias were successfully cultured in vitro, as confirmed by Iba1 staining (Fig. S5a). Then the PPARγ protein level was determined in the microglias of WT or Pdcd4 knockout (Pdcd4−/−) with vehicle or LPS treatment. The data showed the protein level of PPARγ not changed in microglias of Pdcd4 knockout mice, but Pdcd4 deletion increased PPARγ after LPS stimulation for 24 h (Fig. S5b, c). Cycloheximide (CHX) is used to block protein synthesis. Under LPS conditions, the expression of PPARγ rapidly decreased along with CHX in WT derived-microglia, while the phenotype disappeared with the loss of Pdcd4 (Fig. S5d, e). Though Pdcd4 didn’t alter the total PPARγ at baseline, we separated the proteins of cytoplasm and nucleus in the PFC of the mice, and found that nuclear PPARγ was increased in the systemic Pdcd4 knockout mice (Fig. 3a). Meanwhile, the concentrated expression of PPARγ in the nucleus was confirmed in the Pdcd4 deficient microglia at normal or at LPS treatment (Fig. 3b, c). The further immunofluorescent results showed that loss of Pdcd4 improved nuclear PPARγ level (Fig. 3d–f). To examine whether Pdcd4 directly determines PPARγ subcellular distribution, HEK293 cells were transfected GFP tagged-PPARγ with or without Flag tagged-Pdcd4, GFP scatter is on the behalf of PPARγ expression, and the overexpression of Pdcd4 decreased the levels of GFP in the nucleus (Fig. 3g–i). These data suggest that Pdcd4 mediates the subcellular distribution of PPARγ.

### Pdcd4 inhibits PPARγ nuclear expression through interrupting the interaction between PPARγ and Daxx

**Fig. 4:**
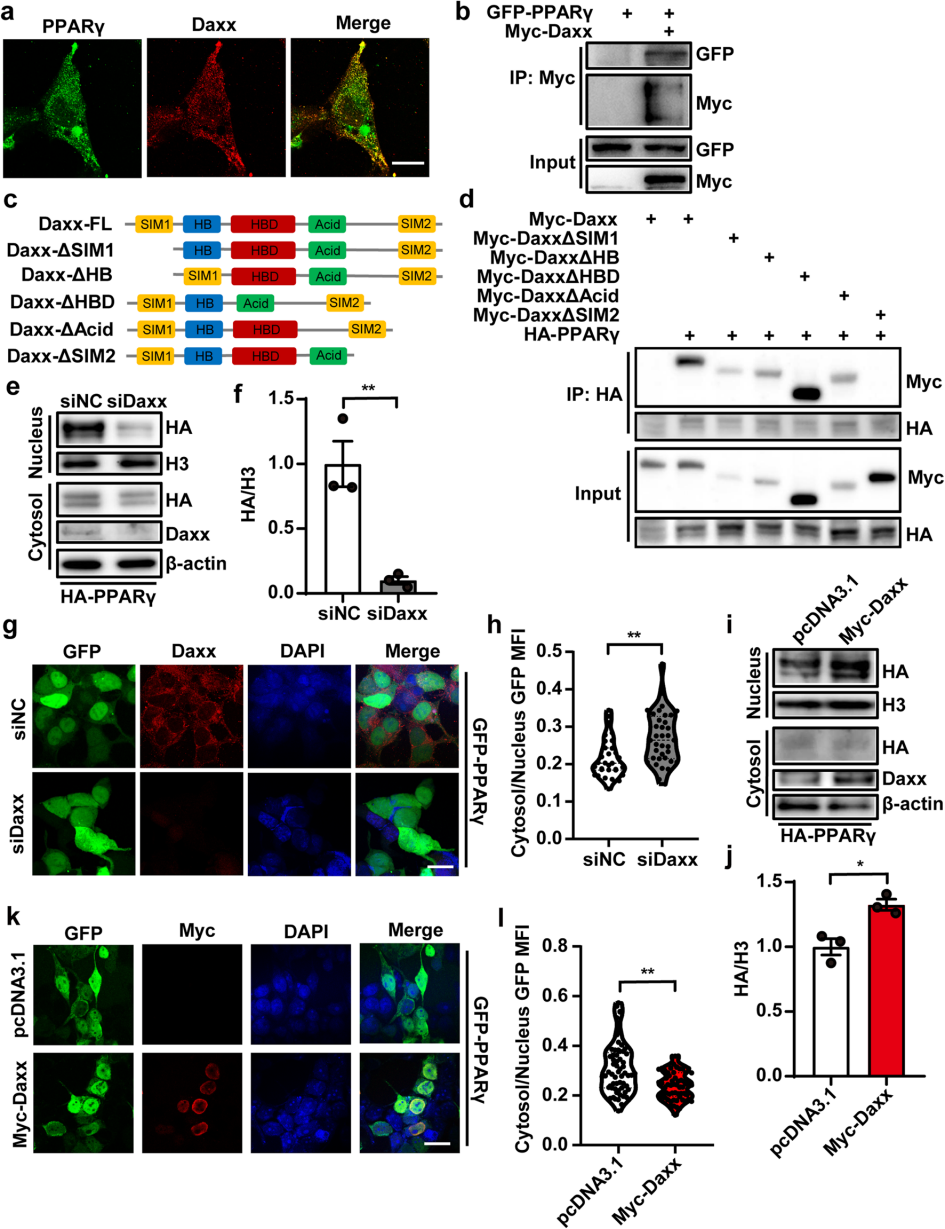
Daxx regulates PPARγ nucleus transportation. **a** Confocal picture showed the colocalization of PPARγ (Green) and Daxx (Red) in the MEF of WT mice; scale bar 20 μm. **b** HEK293 cells were co-transfected with GFP-PPARγ and Myc-Daxx. Immunoprecipitation was performed with the anti-Myc antibody. Immunoblotting was performed with anti-Myc or anti-GFP antibodies. **c** The truncated Daxx plasmid construction. **d** HEK293 cells were co-transfected with HA-PPARγ and Myc-tagged Daxx functional domain deletions. Immunoprecipitation was performed with the anti-Myc antibody. Immunoblotting was performed with anti-Myc or anti-HA antibody. **e**, **f** Nuclear and cytoplasmic proteins were isolated from the HA-PPARγ and siDaxx-transfected HEK293 cells, and determined by SDS-PAGE. N = 3, unpaired two-tailed Student’s t test, **P < 0.01. **g**, **h** HEK293 cells were transfected into GFP-tagged full-length PPARγ by accompany with siNC or siPdcd4 for 24 h, and localizations of GFP were observed by immunofluorescence, and the mean fluorescence intensity of GFP was quantified from n > 30 cells; scale bar 20 μm. Unpaired two-tailed Student’s t test, **P < 0.01. **i**, **j** Nuclear and cytoplasmic proteins were isolated from the HA-PPARγ and Daxx-transfected HEK293 cells, and determined by SDS-PAGE. N = 3, unpaired two-tailed Student’s t test, *P < 0.05. **k**, **l** HEK293 cells were transfected into GFP-tagged full-length PPARγ by accompany with Myc-Daxx for 24 h, and localizations of GFP were observed by immunofluorescence, and the mean fluorescence intensity of GFP was quantified from n > 30 cells; scale bar 20 μm. Unpaired two-tailed Student’s t test, **P < 0.01. The figures represent three independent experiments that yield similar result

Previous study has demonstrated the interaction between Pdcd4 and Fas death domain-associated protein (Daxx) in restraining p53 activity [18]. To explore the mechanisms underlying Pdcd4 regulated PPARγ nuclear transportation, the immunostaining proved that endogenic PPARγ partially co-localized with Daxx in the mouse embryonic fibroblasts (MEF) (Fig. 4a). Next, we co-transfected Myc-tagged Daxx with GFP-tagged PPARγ constructs into HEK293 cells, and the Immunoprecipitation result showed that Daxx could interact with PPARγ (Fig. 4b). In order to identify the specific protein domain of Daxx responsible for its interaction with PPARγ, we generated mutations in Daxx that lack certain domains (Fig. 4c). By immunoprecipitation with the HA antibody, we found SIM2 domain is necessary for the interaction of Daxx with PPARγ, and for SIM2 domain deletion of Daxx diminishing the interaction with PPARγ (Fig. 4d). To test whether Daxx regulates PPARγ nuclear transportation, HA tagged-PPARγ and Daxx siRNA (siDaxx) were co-transfected into HEK293 cells, and the result showed that knockdown of Daxx decreased the level of PPARγ in the nucleus (Fig. 4e, f). Conversely, overexpression of Myc-Daxx up-regulated the nuclear PPARγ expression (Fig. 4i–g). Additionally, GFP-labeled PPARγ was co-transfected with siDaxx or Myc-Daxx into HEK293 cells, and immunostaining for GFP was used to assess the localization of PPARγ within the cells. Consistent with findings from nuclear-cytoplasmic extraction experiments, the expression of Daxx was found to correlate with an increase in nuclear distribution of PPARγ (Fig. 4g, h, k, l), suggesting Daxx regulates PPARγ nucleus transportation.

**Fig. 5:**
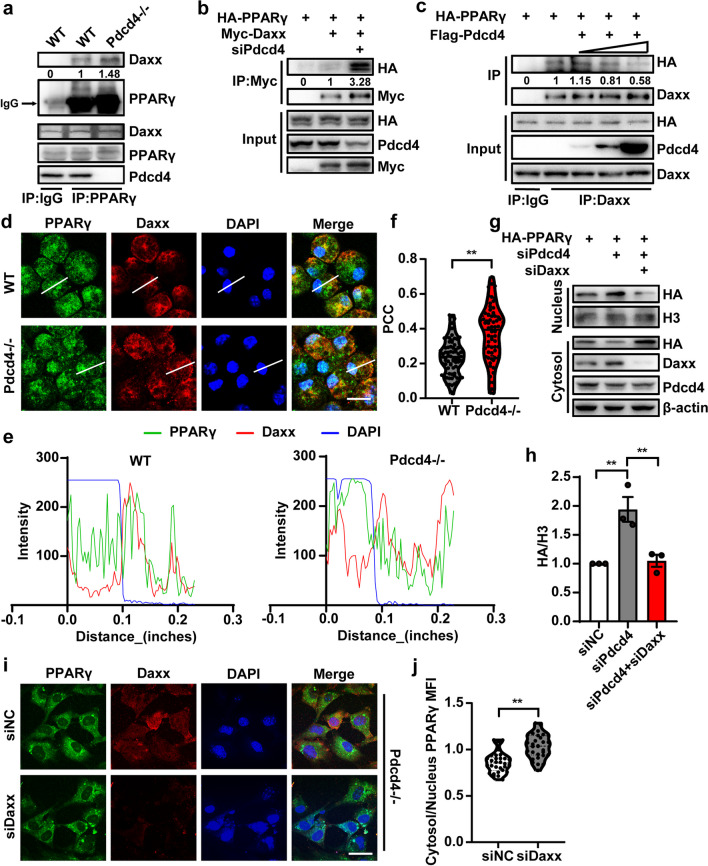
Pdcd4 inhibits PPARγ nuclear expression through interrupting the interaction between PPARγ and Daxx. **a** The PFC of WT or Pdcd4−/− mice was lysis with Immunoprecipitation buffer. Immunoprecipitation was performed with the anti-PPARγ antibody. Immunoblotting was performed with anti-Daxx or anti-PPARγ antibody. **b** HEK293 cells were co-transfected with HA-PPARγ, Myc-Daxx and si Pdcd4. Immunoprecipitation was performed with the anti-Myc antibody. Immunoblotting was performed with anti-Myc or anti-HA antibody. **c** HEK293 cells were co-transfected with HA-PPARγ and Flag-Pdcd4. Immunoprecipitation was performed with the anti-Daxx antibody. Immunoblotting was performed with anti-Daxx or anti-HA antibody. **d**–**f** Confocal picture showed the immunofluorescence of PPARγ (Green) and Daxx (Red) in the peritoneal macrophages of WT or Pdcd4−/− mice; scale bar 20 μm. The co-localized index (Pearson Correlation Coefficient: PCC) was calculated from n > 30 cells. The value of PCC is between − 1 and 1. 1 represents complete positive correlation, − 1 represents complete negative correlation, and 0 represents random relationship (protein A and protein B are randomly distributed and have no correlation). Scale bar 20 μm. Unpaired two-tailed Student’s t test, **P < 0.01. **g**, **h** Nuclear and cytoplasmic proteins were isolated from the HA-PPARγ, siPdcd4 and siDaxx-transfected HEK293 cells, and determined by SDS-PAGE. N = 3, one-way ANOVA and Tukey’s multiple comparisons test (F_2,6_ = 14.78, P < 0.01), **P < 0.01. **i**, **j** siNC or siDaxx was transfected into MEFs from Pdcd4−/− mice for 24 h, and the localization of PPARγ was observed by immunofluorescence, and the mean fluorescence intensity of PPARγ was quantified from n > 30 cells; scale bar 20 μm. Unpaired two-tailed Student’s t test, **P < 0.01. The figures represent three independent experiments that yield similar result

A previous report has found an association between Daxx and Pdcd4 [19], we sought to explore whether Pdcd4 affects the PPARγ-Daxx complex formation. Firstly, the protein level of Daxx was detected by Western blot on the PFC of Pdcd4 microglial knockout mice, and found there have no changes in the groups of LPS administration (Fig. S6a, b). Meanwhile, the endogenous interaction between Daxx and PPARγ was increased in the Pdcd4 knockout peritoneal macrophages compared to the WT control (Fig. 5a). Moreover, knockdown of Pdcd4 could strengthen the interaction between Daxx and PPARγ in cultured HEK293 cells (Fig. 5b). Conversely, the overexpression of Pdcd4 resulted in a decrease in the interaction between PPARγ and Daxx in HEK293 cells (Fig. 5c). Moreover, we performed the immunostaining to observe the subcellular distribution of PPARγ and Daxx in peritoneal macrophage from WT and Pdcd4 deficient mice, and found that Pdcd4 knockout enhanced the colocalization of PPARγ with Daxx and the PPARγ nucleus distribution (Fig. 5d–f). Above all, these results suggest that Pdcd4 interrupts the interaction between PPARγ and Daxx. Furthermore, to explore whether Pdcd4 affects Daxx-mediated PPARγ nuclear transportation, we co-transfected siPdcd4 and siDaxx into HEK293 cells. The findings indicated that the inhibition of Daxx prevented the effect of siPdcd4 on enhancing the nuclear PPARγ protein level (Fig. 5g, h). Similarly, siDaxx also decreased the nucleus PPARγ expression in the MEF from Pdcd4 knockout mice (Fig. 5i, j). Taken together, Pdcd4 hindered Daxx-mediated PPARγ nuclear translocation by disrupting the formation of the complex.

### Pdcd4 regulates PPARγ-related IL-10 transcription

**Fig. 6:**
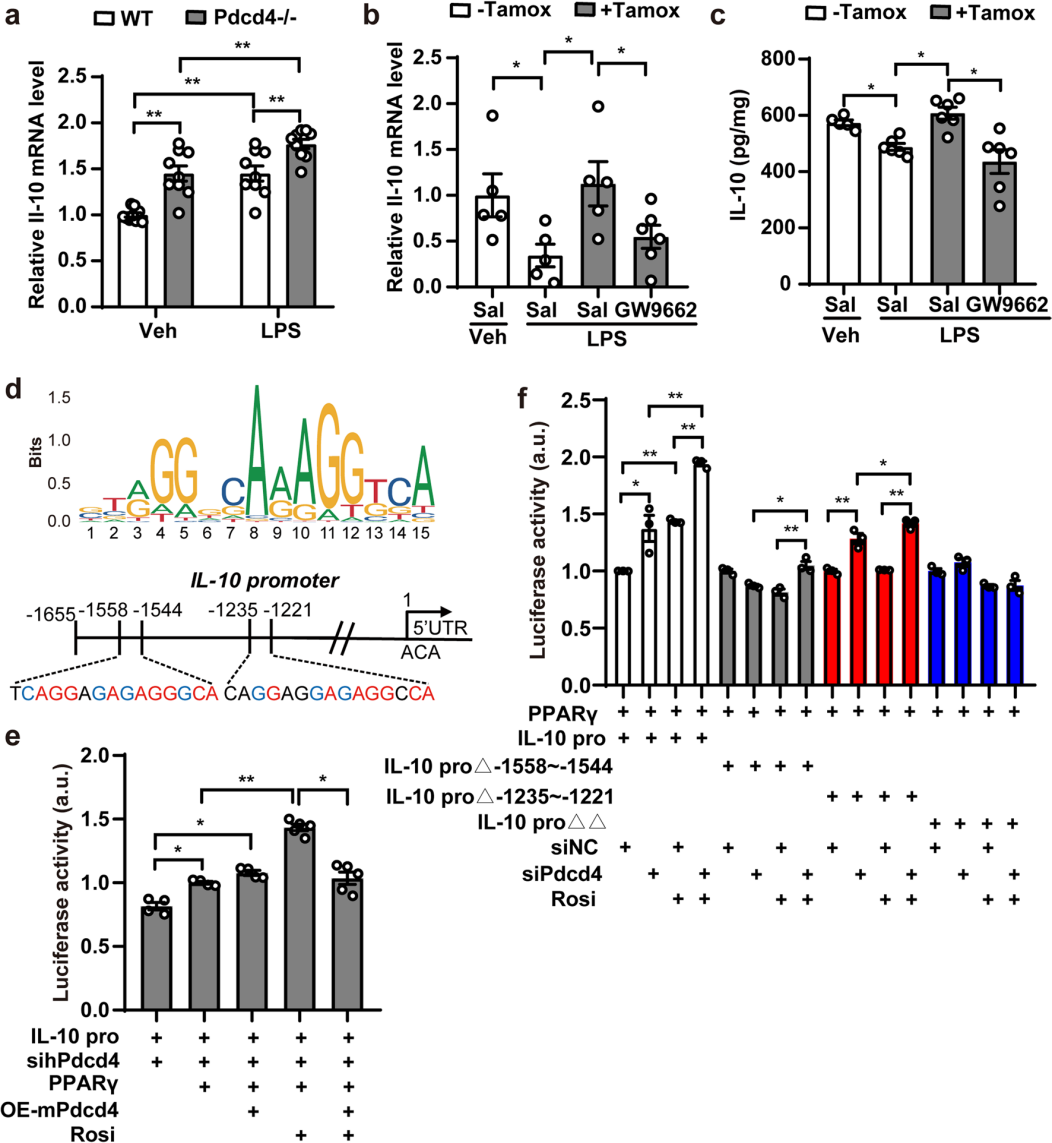
Pdcd4 regulates PPARγ dependent IL-10 transcription. **a** The mRNA changes of il-10 in the primary microglias of the WT or Pdcd4−/− after 1 µg/mL LPS administration for 18 h. n = 3, two-ways ANOVA and Sidak’s multiple comparisons test (Veh vs. LPS: F_1,32_ = 35.08, P < 0.01; WT vs. Pdcd4−/−: F_1,32_ = 35.08, P < 0.01), *P < 0.05, **P < 0.01. **b**, **c** The change of mRNA and protein levels of IL-10 in the PFC of the mcKO mice after LPS and GW9662 administration. n = 5–6 per group, one-way ANOVA and Tukey’s multiple comparisons test (mRNA: F_3,17_ = 3.9, P < 0.05; protein F_3,19_ = 9.429, P < 0.01), *P < 0.05. **d** Schematic of IL-10 upstream regulatory region showing multiple PPAR/RXR response elements. **e** Luciferase assays were performed after transfecting both pGL3-IL10 promoter, siPdcd4 and PPARγ construction by accompany with Pdcd4 overexpression into HEK 293T cells. Renilla luciferase vector was co-transfected for normalization. 24 h post-transfection, cells were stimulated with rosiglitazone (rosi). 2 h post-stimulation, reporter activity was measured. Three independent experiments are shown, mean ± SEM, one-way ANOVA and Tukey’s multiple comparisons test (F_4,17_ = 51.47, P < 0.01). *P < 0.05, **P < 0.01. **f** Luciferase assays were performed after transfecting both pGL3-IL10 promoter or pGL3-IL10 promoter with PPRE deleted-mutations, and PPARγ construction by accompany with siPdcd4 into HEK 293T cells. Renilla luciferase vector was co-transfected for normalization. 24 h post-transfection, cells were stimulated with rosiglitazone (rosi). 2 h post-stimulation, reporter activity was measured. Three independent experiments are shown, mean ± SEM, one-way ANOVA and Tukey’s multiple comparisons test (F_15,32_ = 60.92, P < 0.01). *P < 0.05, **P < 0.01

We next explored the mechanism underlying microglial Pdcd4 deficiency-induced antidepressant effect in neuroinflammation-associated depression. Multiple cytokines have been regulated by PPARγ, such as Tnf-α, Inos, Il-1β, Ccl2, and Il-10. Initially, we assessed the mRNA expression levels of cytokines in microglia cells, revealing an up-regulation of Il-10 expression in the Pdcd4−/− group. However, Pdcd4 have no effect on the expression of Tnf-α, Inos, Il-1β under normal or LPS stimulation conditions (Fig. 6a; Fig S7a). To prove whether Pdcd4-regulated IL-10 expression was mediate by PPARγ, the cytokines expression in the PFC of the microglial Pdcd4 knockout mice was detected. The data showed that Tnf-α, Inos, Il-1β, Ccl2 and Il-10 were increased or decreased in the LPS-treated control mice but not in the Pdcd4 conditional deletion mice. Moreover, GW9662 treatment significantly reduced the mRNA and protein expression of IL-10 in the microglial Pdcd4 knockout compared to the saline-treated group under LPS injection conditions, but it did not affect the mRNA expression of Tnf-α, Inos, Il-1β, and Ccl2 (Fig. 6a, b; Fig. S7b–e). These data suggested the involvement of Pdcd4 in the PPARγ/IL-10 axis. Although IL-10 as a neuroinflammatory factor is involved in depression, the transcriptional regulatory mechanism of IL-10 in the microglia has not been elucidated. As previously reported, analysis of the promoter in the IL-10 gene revealed multiple PPARγ response elements (PPREs) (Fig. 6c). Next, we performed the luciferase reporter assay in HEK293 cells to detect the effect of Pdcd4 on IL-10 expression. The activation of PPARγ by the agonist rosiglitazone (Rosi) markedly enhanced the transcription of IL-10; conversely, the overexpression of Pdcd4 reversed this effect (Fig. 6d). While rosiglitazone has been shown to enhance IL-10 transcription, the knockdown of Pdcd4 results in a heightened exacerbation of this effect. However, the stimulation of IL-10 transcription by rosiglitazone was found to be significantly reduced in the absence of PPREs within the IL-10 promoter (Fig. 6e). Collectively, these data indicated an important role for Pdcd4/PPARγ axis in IL-10 expression.

### Microglial Pdcd4 knockout resolves LPS-induced depressive behavior by rescuing IL-10

**Fig. 7:**
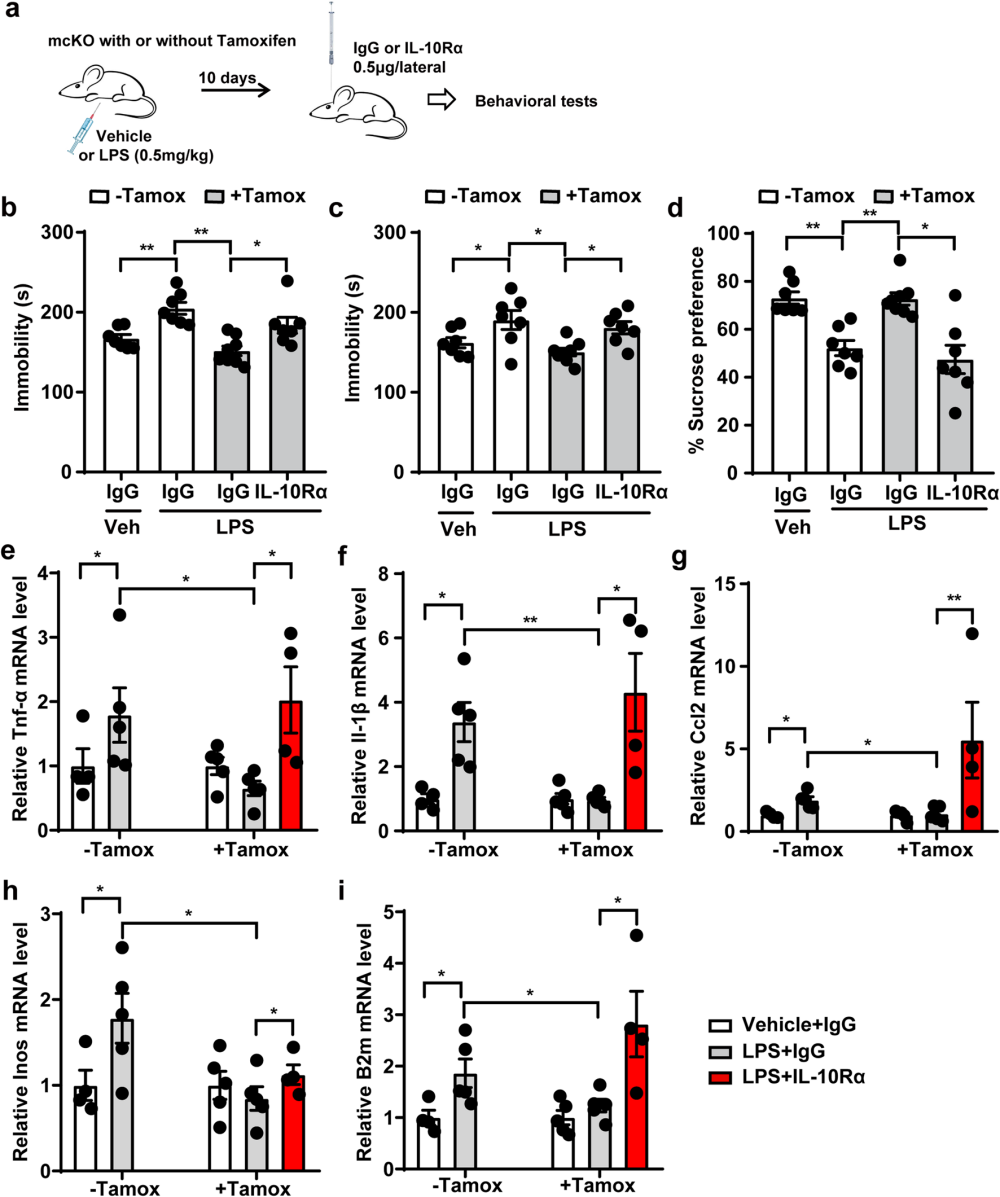
Microglial Pdcd4 knockout resolves LPS-induced depressive behavior by rescuing IL-10. **a** Schematic diagram of IL-10Rα-induced mcKO mice. **b** Immobility time in TST (F_3,25_ = 10.02, P < 0.01), **c** immobility time in FST (F_3,25_ = 5.363, P < 0.01), and **d** sucrose consumption in SPT (F_3,25_ = 12.88, P < 0.01). One-way ANOVA and Tukey’s multiple comparisons test, *P < 0.05, **P < 0.01. **e**–**i** The change of mRNA levels of TNFα, IL-1β, CCL2, iNOS, B2M. n = 4–5 per group, unpaired two-tailed Student’s t test, *P < 0.05, **P < 0.01

To investigate the potential role of IL-10 in Pdcd4-associated depressive-like behavior, we administered a neutralizing IL-10 antibody, IL-10Rα, via intracerebroventricular injection to Pdcd4 mcKO mice prior to conducting behavioral assessments (Fig. 7a). We found that IL-10Rα treatment significantly blocked the decreased immobility in TST and FST trials and the improved preference to sucrose in SPT in the LPS treated mcKO mice (Fig. 7b–d). Meanwhile, we confirmed that IL-10Rα treatment did not worsen depressive-like behavior in WT mice injected with LPS (Fig. S4a, b). Finally, The expression of Tnf-α, Il-1β, Ccl2, Inos, and B2m was dramatically elevated in the PFC of the IL-10Rα-injected Pdcd4 microglial knockout mice in response to LPS (Fig. 7g–k). In summary, the data indicates that the increased expression of IL-10 plays a role in the antidepressant properties observed in microglial Pdcd4 knockout mice following exposure to LPS.

## Discussion

Neuroinflammation is thought to drive changes in neurotransmitters and neurocircuits that lead to major depressive disorder (MDD). In this article, we provide direct evidence that microglial Pdcd4 mediates neuroinflammation-associated microglia activation and consequent depressive-like behaviors in mice by interrupting the PPARγ mediated IL-10 transcription.

Firstly, we confirmed Pdcd4 is involved in inflammatory-related depression via regulating microglia activation. Regardless of the devasting role of neuronal Pdcd4 in chronic restraint stress (CRS)-induced depression [16, 17], we found microglia Pdcd4 knockout protect mice from LPS-induced depressive-like behaviors. LPS challenge, mimicking an acute inflammatory response, stimulated peripheral immunity, and subsequently evoked neuroinflammation and depressive-like behaviors [20]. Mechanically, LPS stress caused more microglia activation and stronger cytokines expression than CRS, resulting in a significant difference between the LPS and CRS models in aspects of intensity and duration of stress. Though the prefrontal cortex, hippocampus, striatum, and hypothalamus are well-known brain areas that have a relationship with depression, multiple studies have referred to significant cytokines’ expression and microglia activation in the PFC rather than in the HIP [21]. Our study suggests that the expression profile of Pdcd4 may play a role in neuroinflammatory responses across various brain regions under LPS conditions. Western blot and RT-PCR techniques are limited in their ability to detect changes in Pdcd4 at the single-cell level. Interestingly, pathological staining revealed an upregulation of Pdcd4 in both microglia and non-microglia cells in the prefrontal cortex. These findings may be attributed to variations in cell types present in different brain regions [22]. Understanding the upstream regulatory mechanism of Pdcd4 expression can reveal how different brain regions respond to stress. Recently report found LPS required the NF-κB signaling activation to boost Pdcd4 expression in microglia, perhaps discovering the NF-κB signaling response pattern during the LPS challenge will help us to answer that question [23]. In summary, we discovered that microglial Pdcd4 plays a role in LPS-induced depressive behavior, highlighting the various functions of Pdcd4 in nerve cells and brain regions, particularly its immunoregulatory role in the PFC during neuroinflammation-related depression.

Secondly, we discovered the involvement of the Pdcd4/PPARγ axis in depressive-like behavior. A previous study has referred that lncRNA-H19 facilitated Pdcd4 expression in microglia via sponging miR-21, which initiated the Ischemia–reperfusion (I/R)-induced inflammation [24]. However, the specific mechanism underlying Pdcd4-regulated neuroinflammation in depression is still unknown. RNA-sequencing analysis elucidates the regulatory role and potential mechanism of Pdcd4 in microglia, with enrichment of the KEGG pathway in thermogenesis and oxidative phosphorylation. Mitochondrial dysfunction is recognized as a contributing factor to emotional disorders, while recent research indicates impaired energy metabolism in individuals with depression, specifically affecting mitochondrial oxidative phosphorylation in glutamatergic neurons [25, 26]. Therefore, abnormal energy regulation of synapses is a potential mechanism for emotional disorders [27]. Our prior studies have shown that Pdcd4 plays a detrimental role in metabolic disorders by inhibiting the transition of white adipose tissue to beige adipose tissue and facilitating the formation of stress granules [28, 29]. Further exploration is needed to determine whether Pdcd4 plays a role in mitochondria function, which is implicated in cell metabolism and may contribute to the development of depression. PPARγ is one of the three isoforms of PPARs, and is activated by thiazolidinediones such as pioglitazone and is applied for insulin resistance treatment. Previous reports have demonstrated that neuronal PPARγ directly mediated stress-induced emotional disorders and PPARγ agonists have an antidepressant effect, suggesting the essential role of PPARγ in depression [11]. Based on the enrichment of PPARγ expression in microglia, PPARγ also has anti-inflammatory properties [30]. In this study, we observed that the deletion of microglial Pdcd4 resulted in the inhibition of LPS-induced microglial activation by up-regulating PPARγ and enhancing nuclear transportation. Previous studies have established a causal relationship between PPARγ protein stability and its subcellular localization within the nucleus, as cytosolic PPARγ is rapidly degraded through ubiquitin–proteasome-mediated mechanisms [31]. While several E3 ubiquitin ligases, such as NEDD4-1, TRIM25, FBXO9, MKRN1, CUL4B, and FBXO4, have been implicated in the regulation of PPARγ protein levels, the mechanism of LPS-mediated protein degradation of PPARγ remains poorly understood [32–37]. In conclusion, microglial PPARγ is necessary for neuroinflammatory response.

Thirdly, we illustrated Daxx as an adaptor, which is critical for PPARγ cytosol-nuclear shuttle. Evidence suggests that PPARγ is exported from the nucleus in a MEK-dependent manner through serine 112 phosphorylation [38, 39]. However, the specific process by which PPARγ nucleus translocation in various cell types, particularly microglia, has yet to be fully elucidated. Herein, we found Daxx is recruited by PPARγ in a dynamic manner, leading to the translocation of PPARγ into the nuclear compartment. The interaction between Pdcd4 and Daxx results in a reduction in the half-life of Daxx, ultimately disrupting the formation of the PPARγ/Daxx complex [19]. Therefore, Pdcd4 is a switch for Daxx-mediated PPARγ nuclear translocation, and Pdcd4 deletion stabilizes PPARγ in the nuclei. As previously mentioned, Daxx functions as a co-repressor in macrophages, inhibiting the expression of NF-κB-targeted pro-inflammatory genes through its interaction with histone deacetylases and DNA methyltransferases [40]. Hence, we provided a potential mechanism of PPARγ for inhibiting inflammation via Daxx-related DNA epigenetic modification. Additional research is required to elucidate the involvement of PPARγ in the Daxx-mediated induction of proinflammatory gene transcription.

Finally, our data demonstrated the antidepressant role of IL-10. Research on IL-10 knockout mice found an increase in depressive-like behaviors that were reversed with IL-10 injection [41]. In addition, the drugs for depression treatment, such as Fluoxetine and Sertraline, have displayed anti-inflammatory properties, including IL-10 up-regulation [3]. However, further research is needed to elucidate the mechanisms underlying the observed changes in relation to IL-10. Our study indicates that the inhibition of PPARγ leads to a suppression of IL-10 expression in microglia during depression. By demonstrating the ability of pioglitazone to improve depression-like behaviors through the promotion of a neuroprotective microglial phenotype, we have provided direct evidence supporting the anti-inflammatory function of PPARγ in enhancing mRNA transcription via binding to the IL-10 gene promoter. Overall, we emphasized the therapeutic role of IL-10 for depression.

In conclusion, to the limitation of traditional therapy of depression, we are urged to find the new pathological mechanism of the disease. Our findings show that neuroinflammatory reaction, including microglia activation and cytokines secretion is integrated into depressive-like behaviors via activation of Pdcd4 mediated PPARγ/IL-10 axis.

## Methods

### Animals

The littermate wild type (WT) and Pdcd4-deficient male mice in this study have been previously described. Also, LoxP-flanked Pdcd4 mice were generated by Biocytogen Co., Ltd (Beijing, China) using CRISPR/Cas 9 which has been referred to before [16]. Briefly, by crossing with Cx3cr1-CreERT2 (Jax stock no. 021160), we get microglial conditional Pdcd4 knockout mice, and Pdcd4^fl/fl^ mice are used for littermate control. For inducing gene knockout, tamoxifen (50 mg/kg, ig) diluted into corn oil is treated to conditional knockout or control mice at 56 days after birth. Male mice are utilized in behavioral and biochemical experiments. All animal experiments and protocols are approved by the Animal Care and Utilization Committee of Shandong University.

### LPS-induced depressive behavior

Male C57BL6/J mice (2 months) are intraperitoneally injected with LPS dissolved in sterile 0.9% saline at 0.5 mg/kg for 10 days. Saline or LPS injection is administered between 09:00 and 09:30 a.m. daily for 10 days. Regents used in animal experiments were list in table below.

**Table:** 

Regent	Manufacturer	Catalog	Usage
Lipopolysaccharide 055:B5	Sigma Aldrich	L2880	ip. 0.5 mg/kg
GW9662	Selleck	S2915	ip. 1 mg/kg
Mouse IL-10Rα antibody	R&D	AF-474	icv. 0.5 μg
Tamoxifen	Sigma Aldrich	T5648	ig. 50 mg/kg
Rosiglitazone	Sigma Aldrich	R2408	10 μM
Cycloheximide	MCE	HY-12320	50 μM

### Behavioral tests

#### Open field test

Mouse is placed in the area (40 × 40 × 35 cm, L × W × H) with 60 lux lighting for 10 min. A SMART tracking system (Panlab, DC, USA) is used to record the movement of mice and analyze the traveled distance.

#### Elevated plus maze

The apparatus sets to a height of 50 cm beyond the ground and has black stainless steel with two closed and open arms (30 × 5 × 10 cm walls or no wall). Use LED lights to control the brightness of the light. To simulate the sense of security of animals in natural environments, the closed arm brightness is set 100 lux. Mouse is placed into the center platform facing an open arm and a video tracking system scores the time spent in open arms.

#### Tail suspension test

Each mouse is hung to a grid bar of 30 cm which is the height from the ground by the tail with tape. Then the test is video-captured for 6 min. The immobile time is counted, and immobility is defined as the absence of escape-orientated movement.

#### Forced swimming test

The mouse is individually placed in a glass cylinder (25 × 18 cm diameter) which contains water (15 cm height, 22 ± 0.5 °C). The experiments are videotaped by a camera for tracking mice’s reactions and scored the latency to immobility. The time of immobility is determined by the absence of motion except for slight actions to maintain the head above the water.

#### Sucrose preference test

Mice are habituated to drink sucrose solution (1%) over 2 days. After deprivation of water for 22 h, mice are given free access to two bottles separately containing water or 1% sucrose solution for 2 h. At the next day’s test, the site of the two bottles is exchanged to avoid a side bias. Finally, the fluid consumption is indicated by the weight loss of the bottles. Sucrose preference is calculated as follows: Sucrose preference (%) = sucrose intake/total fluid consumption × 100%.

### Primary microglias isolation

Coat T-75 culture flask with 10 μg/mL PDL for 2 h, and wash the flask bottom with sterile water 3 times before use. Postnatal 1–3 days WT or Pdcd4−/− mouse were anesthetized, and brains were rapidly dissected and placed into ice-cold PBS. Brain material was cut off small pieces, and placed into 15 mL tube with 0.25% Trypsin–EDTA for 20 min at 37 °C. Aspirate the supernatant and resuspend the pellet with 5 mL warmed DMEM/F12 containing 10%FBS, 100 IU/mL penicillin and 100 μg/mL streptomycin, after that centrifuge the 15 mL tubes at 400×*g* for 5 min. Plate the cells into coated T-75 flask and change the culture medium the next day to remove cell debris. In 5–7 days, microglia and some oligodendrocytes grow on the top of astrocytic layer. Tapping the flasks vigorously on the bench top, the floating cells were collected in culture media. The microglial cells are ready to use the next day.

### Cell culture

HEK293 is purchased from the National Collections of Authenticated Cell Cultures (Shanghai, China). Mouse embryonic fibroblasts (MEFs) of Pdcd4 conventional knockout or littermate mice were prepared from E13.5 embryos. The 6–8 weeks-old mice are injected (i.p.) with 6% broth starch solution, and 3 days later peritoneal cells are collected. All media are Dulbecco’s modified eagle medium (DMEM) supplemented with 10% FBS (Cat. 10099-141, Gibco, USA), 100 IU/mL penicillin, and 100 μg/mL streptomycin (Cat. 03-031-1B, Biological Industries, Israel). Cells were all cultured in a humidified cell incubator with an atmosphere of 5% CO^2^ at 37 °C.

### Immunohistochemistry

A mouse heart is instilled in vivo with 4% polyformaldehyde after anesthesia by 5% chloral hydrate (7.5 mL/kg, i.p). The brain is harvested and embedded into OCT, after then, it is sectioned 40 μm thick. For the immunofluorescence, slides are incubated in 0.4% TritonX-100 diluted donkey serum to 10% for 1 h. Anti-Iba1 or anti-Pdcd4 antibody is used to slide incubation overnight at 4 °C, after that, those are washed with PBS three times. The secondary antibody incubated the slides at room temperature for 1 h. Washing in PBS three times, slides are mounted with cover glass. All the images are captured with an Olympus VS120 microscope (Tokyo, Japan). Pictures are analyzed by NIH Image J.

### Real-time PCR

Mice are sacrificed immediately after behavioral tests. The hippocampus, prefrontal cortex, and hypothalamus are collected using a mouse brain slicer. Total RNA is extracted by Trizol reagent (Tiangen, Beijing, China) following the manufacturer’s instructions. Then, the purified total RNA (1000 ng) is reversed to cDNA by using the cDNA Synthesis Kit (TAKARA, Tokoyo, Japan). Real-time PCR primers were listed in table below.

**Table:** 

Gene name	Species	Sequence
Pdcd4	Mus	Forward: 5′ AAACAACTCCGTGATCTTTGTCCA 3′ Reverse: 5′ TCAGGTTTAAGACGGCCTCCA 3′
Pparγ	Mus	Forward: 5′ GTACTGTCGGTTTCAGAAGTGCC 3′ Reverse: 5′ ATCTCCGCCAACAGCTTCTCCT 3′
Tnfα	Mus	Forward: 5′ CCCTCACACTCAGATCATCTTCT 3′ Reverse: 5′ GCTACGACGTGGGCTACAG 3′
Il-1β	Mus	Forward: 5′ GCAACTGTTCCTGAACTCAACT 3′ Reverse: 5′ ATCTTTTGGGGTCCGTCAACT 3′
Ccl2	Mus	Forward: 5′ AGGTGTCCCAAAGAAGCTGT 3′ Reverse: 5′ GACCTTAGGGCAGATGCAGTT 3′
Il-10	Mus	Forward: 5′ GCCAGAGCCACATGCTCCTA 3′ Reverse: 5′ GATAAGGCTTGGCAACCCAAGTAA 3′
B2m	Mus	Forward: 5′ CCCGCCTCACATTGAAATCC 3′ Reverse: 5′ GTCTCGATCCCAGTAGACGG 3′
iNos	Mus	Forward: 5′ GTTCTCAGCCCAACAATACAAGA 3′ Reverse: 5′ GTGGACGGGTCGATGTCAC 3′
β-Actin	Mus	Forward: 5′ CAACTTGATGTATGAAGGCTTTGGT 3′ Reverse: 5′ ACTTTTATTGGTCTCAAGTCAGTGTACAG 3′

### Nuclear and cytoplasmic extraction

The experiment follows the manufacturer’s instruction for NE-PER Nuclear and Cytoplasmic Extraction kit (Cat. 78833, Thermo Fisher, USA). Briefly, cultured cells are harvested with trypsin–EDTA and then centrifuge at 500×*g* for 5 min. After washing cells with PBS, we add ice-cold CER I to the cell pellet and incubate on ice for 10 min, after that, add ice-cold CER II to the tube and centrifuge that for 5 min at maximum speed. Next, suspend the insoluble fraction in ice-cold NER for 40 min, and centrifuge the tube at maximum speed for 10 min. Prepare the supernatants for Western blot.

### Western blot and ELISA

Mice brain tissue or cultured cell is homogenized in lysis buffer with protease inhibitors and ready for western blot and ELISA. The IL-10 level of the mouse brain is determined by IL-10 ELISA Kit according to the instructions (Cat. 431414, Biolegend, USA). All the primary and secondary antibodies have been listed in Table.

**Table:** 

Name	Manufacturer	Catalog	Source	Application and dilute
Pdcd4	CST	9535S	Rabbit	WB 1:2000 ICC 1:500
PPARγ	Santa Cruz	sc-7273	Mouse	WB 1:1000 ICC 1:200 IP 1:100
Daxx	Sigma Aldrich	D7801	Rabbit	WB 1:1000 ICC 1:250 IP 1:200
GFP	Invitrogen	A11122	Rabbit	WB 1:3000
HA	Elabscience	E-AB-40523	Rabbit	WB 1:2000
Myc	Bethly	A190-105A	Rabbit	WB 1:3000
Histone-H3	Proteintech	17168-1-AP	Rabbit	WB 1:1000
β-Actin	Beyotime	AA128	Mouse	WB 1:2000
Iba1	WAKO	019-19741	Rabbit	IHC 1:500
Iba1	Abcam	ab5076	Goat	IHC 1:500
Anti-Myc magnetic beads	Bimake	B26301	Mouse	IP 1:20
APC anti-CD45	Biolegend	157605	Mouse	FCM 0.5 µL
PE anti-CD11b	Biolegend	101207	Rat	FCM 0.5 µL
HRP anti-Rabbit IgG	Beyotime	A2080	Goat	WB 1:5000
HRP anti-Mouse IgG	Beyotime	A2016	Goat	WB 1:5000
Alexa Fluor™ 594 anti-Rabbit IgG	Thermo Fisher	A21207	Donkey	IHC 1:500 ICC 1:500
Alexa Fluor™ 488 anti-Mouse IgG	Thermo Fisher	A21202	Donkey	IHC 1:500 ICC 1:500

### Flow cytometry

A mouse heart is instilled in vivo with PBS after anesthesia by 5% chloral hydrate (7.5 mL/kg, i.p). Collect the brain and cut it into 1–2 mm^3^ pieces with small scissors. Transfer the sample to a 50 mL conical using a 10 mL digestion cocktail per brain at room temperature for 15 min. Resuspend the pellet in 37% percoll, and then on top of that layer slowly pipette 4 mL of 30% percoll, followed by 2 mL of HBSS. Centrifuge gradient 40 min at 200×*g*. Collect the 70–37% interphase into a clean 50-mL conical tube, and wash it with HBSS 3 times. Obtained single-cell suspension is labeled by using antibodies, and data are obtained on Cytoflex S (Backman, USA).

### Immunocytochemistry

Cultured cells are fixed with 4% paraformaldehyde. For the immunofluorescence, slides are incubated in 0.4% TritonX-100 diluted donkey serum to 10% for 1 h. Primary antibodies are used for samples incubation overnight at 4 °C, after that, those are washed with PBS three times. Secondary antibodies incubate the slides at room temperature for 1 h. Then, the samples are washed three times with PBS and mounted on slides in Prolong Gold medium (Invitrogen, USA). All the images were captured with a Zeiss LSM880 confocal microscope fitted with a 63× oil-immersion objective lens.

### Immunoprecipitation

Cell proteins are extracted with TNE buffer (10 mM Tris, 150 mM NaCl, 1 mM EDTA, 1% NP-40, 10% glycerol, and protease inhibitors). The cell lysates are precipitated with antibody or Protein A/G beads overnight at 4 °C. The beads are rinsed 4 times with the TNE buffer.

### Luciferase assay

Luciferase activity is measured with a dual luciferase assay system (Cat. E1910, Promega, USA) in HEK293 cells with siRNA or overexpression plasmids, and the readout is determined by using a microplate luminometer (Centro LB 960, Germany).

### RNA-Seq

A total of 3 μg RNA was isolated using TRIzol, and sequencing libraries are generated using NEBNext UltraTM RNA Library Prep Kit for Illumina (NEB, Ipswich, MA, USA). RNA sequencing (RNA-seq) was performed on an Illumina HiSeq 4000 platform according to the manufacturer’s instructions and using a paired-end approach with 150-bp reads. Raw sequence read files are quality checked by FastQC software.

### Statistical analysis

Data are displayed as the mean ± SEM and analyzed by using GraphPad Prism 8.0 (GraphPad Software, CA, USA). Normal distributions are compared by using the Kolmogorov–Smirnov test. One-way ANOVA followed by Tukey’s post hoc test and unpaired Student’s t-tests are performed. Statistical tests were two-sided and *p < 0.05, **p < 0.01 was regarded as statistically significant. All data points provided are biological replicates and represent n.

### Supplementary Information

Supplementary Material 1.

## Data Availability

Data supporting the present study are available from the corresponding author upon reasonable request.
