# Supplementary material for: Microglial Pdcd4 deficiency mitigates neuroinflammation-associated depression via facilitating Daxx mediated PPARγ/IL-10 signaling
Source: J Neuroinflammation. 2024 May 31;21:143. doi: 10.1186/s12974-024-03142-3 (PMC11141063; doi:10.1186/s12974-024-03142-3)
Supplement: Supplementary file 1 — Supplementary Material 1. [file 12974_2024_3142_MOESM1_ESM.docx]

**Supplementary Figure 1. LPS-treatment induced depression-like behavior in mice.** (a) Schematic diagram of behavioral test of LPS-induced mice. (b) Immobility time in TST, (c) immobility time in FST under basal or LPS conditions. N=9 per group, unpaired two-tailed Student’s t test, *P<0.05, **P<0.01.


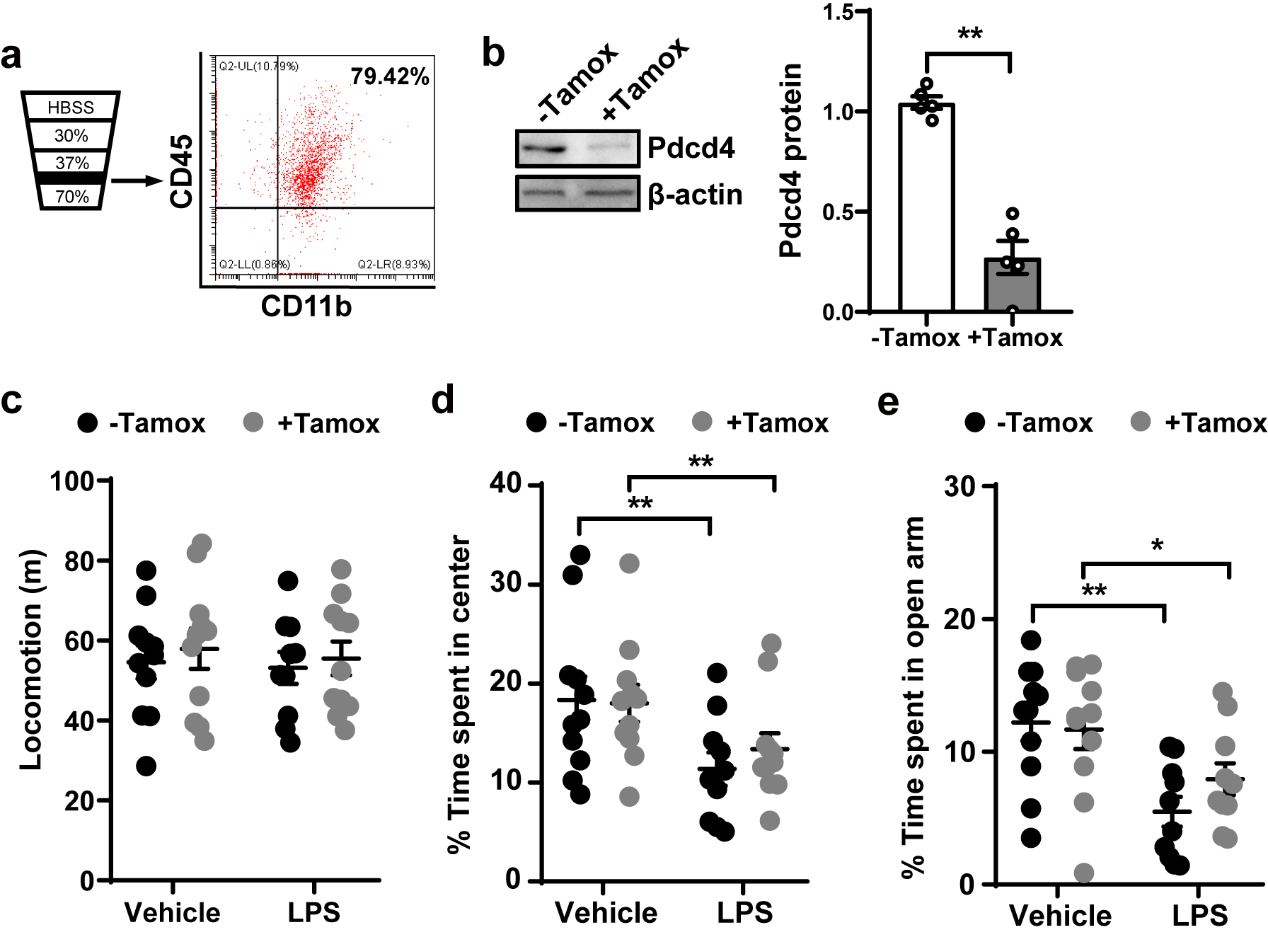


**Supplementary Figure 2. Microglial Pdcd4 knockout couldn’t reverse LPS-induced anxiety-like behavior.** (a) Representative flow cytometry of CD45+/CD11b+ cells from the PFC of WT mice. (b) The protein levels of Pdcd4 in the purified microglia derived from the whole brain of mcKO mice with or without tamoxifen, n=5 per group, unpaired two-tailed Student’s t test, **P<0.01. (c) The moved distance of these mice in the open field test (Veh vs. LPS F_1,39_=0.1914, P=0.66; -Tam vs. +Tam F_1,39_=0.422, P=0.51). (d) The time (%) spent in the center of these mice in the open field (Veh vs. LPS F_1,39_=9.3, P<0.01; -Tam vs. +Tam F_1,39_=0.194, P=0.66). (e) The time (%) spent in the open arm of these mice in an elevated plus maze (Veh vs. LPS F_1,39_=15.96, P<0.01; -Tam vs. +Tam F_1,39_=0.53, P=0.46). Two-ways ANOVA and Sidak’s multiple comparisons test, *P<0.05, **P<0.01.


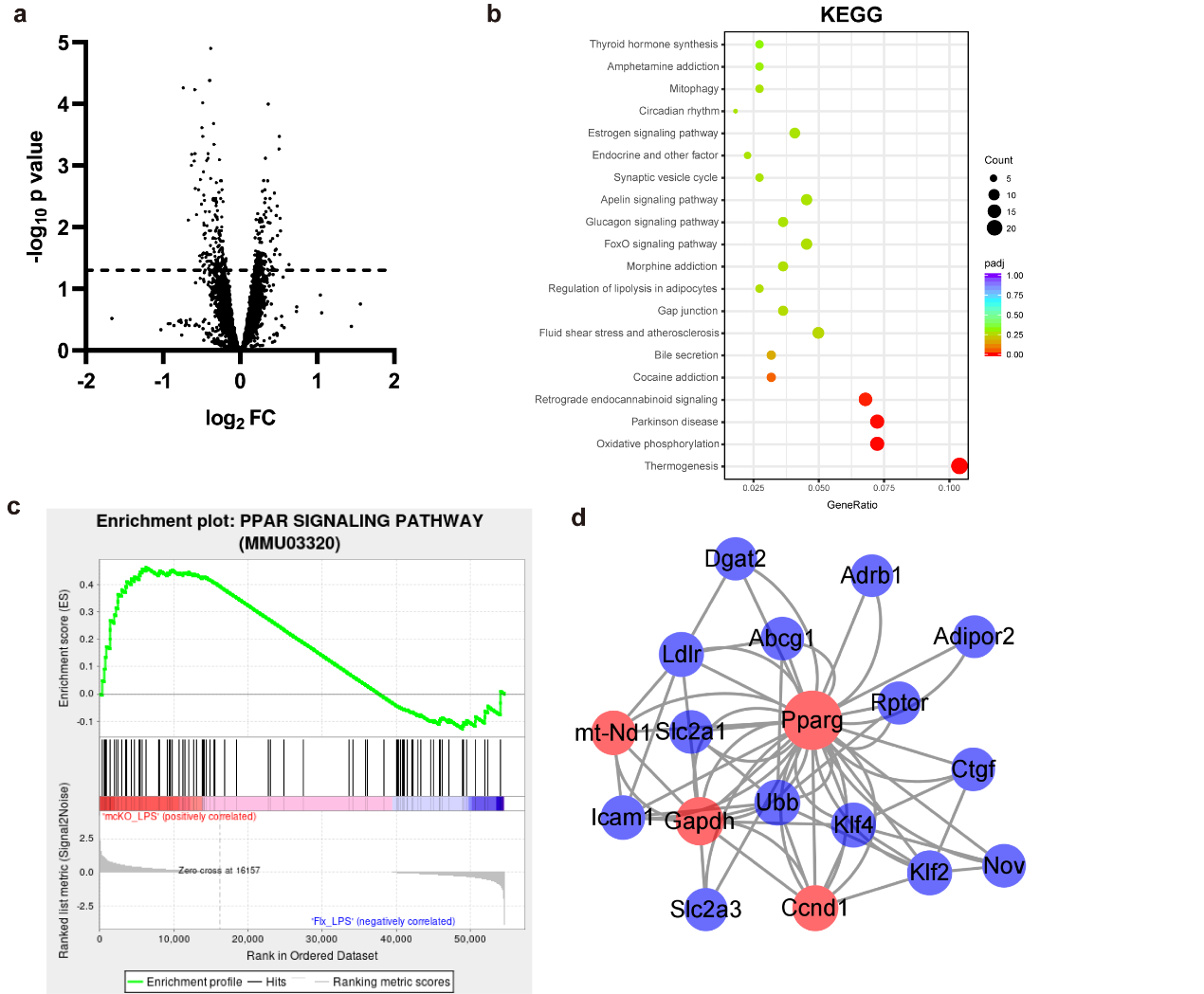


**Supplementary Figure 3. Transcriptomic analysis identified that the gene expression profile was altered in the PFC of microglial Pdcd4 knockout mice.** (a) Volcano plot indicated the scatting of the differential expressed genes between mcKO mice and their littermates. (b-c) KEGG pathway analysis of differentially expressed genes (DEGs) between mcKO mice and their littermates. Size and color of the bubble represent amount of DEGs enriched in the pathway and enrichment significance, respectively. (d) Identification of hub genes from the PPI network. The node color changes from red to blue in ascending order according to log2FC.


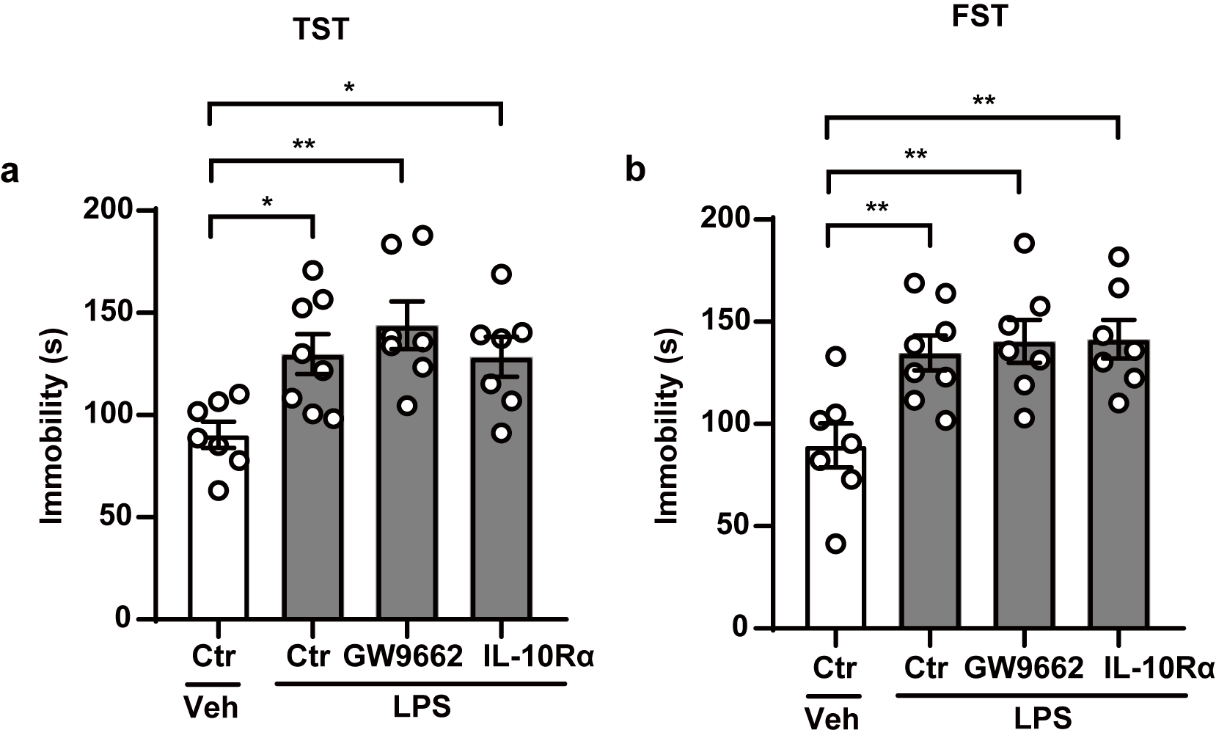


**Supplementary Figure 4. The administration of GW9662 and IL-10Rα didn’t exacerbate depressive-like behavior in the neuroinflammation model.** (a) Immobility time in TST (F_3,25_=5.538, P<0.01), (b) immobility time in FST under basal or LPS conditions (F_3,25_=6.288, P<0.01). n=7-8 per group, one-way ANOVA and Tukey’s multiple comparisons test, *P<0.05, **P<0.05.


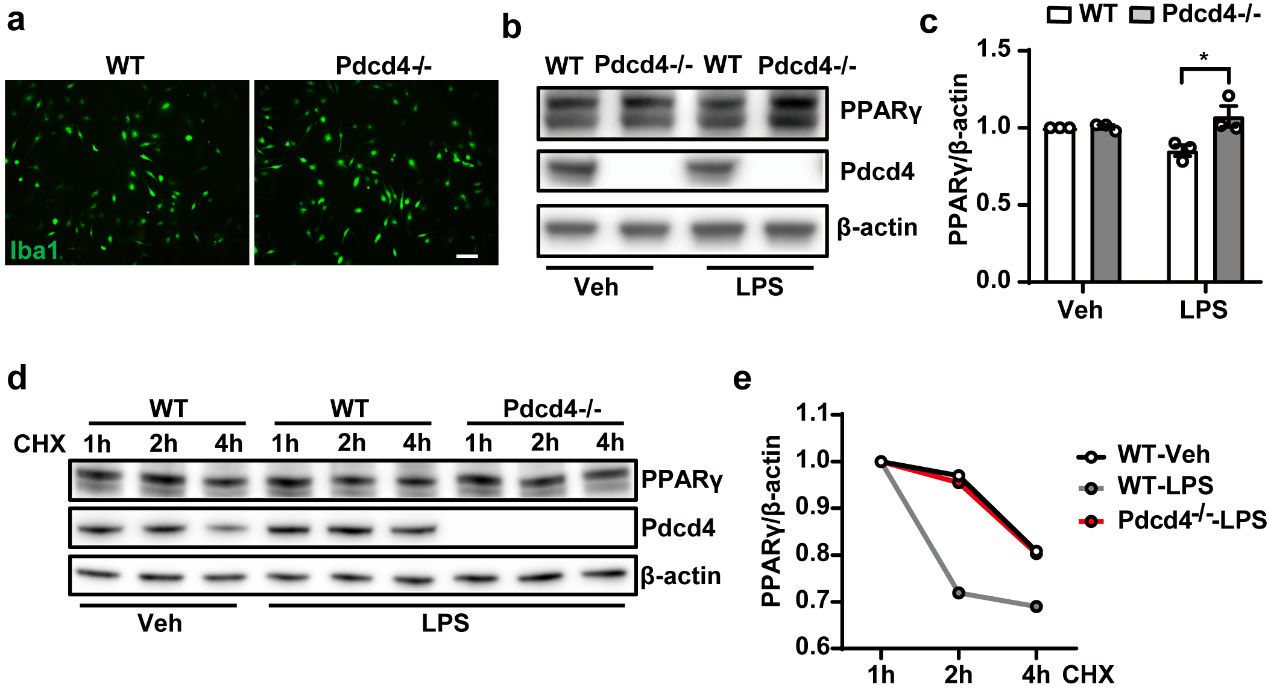


**Supplementary Figure 5. Pdcd4 has no effect on PPARγ expression.** (a) Iba1 antibody maps the purity of isolated microglia from mice brain, scale bar=20μm. (b-c) The protein level of PPARγ in the microglia of WT or Pdcd4-/- with vehicle or 1ug/ml LPS for 24h. n=3 per group, two-ways ANOVA and Sidak’s multiple comparisons test (Veh vs. LPS F_1,8_=1.059, P=0.33; WT vs. Pdcd4-/- F_1,8_=8.76, P<0.01), *P<0.05. (d-e) WT or Pdcd4-/- derived-primary microglias were treated with 1ug/ml LPS or Veh for 24h, after that 50μM Cycloheximide (CHX) were added for 1h, 2h or 4h.


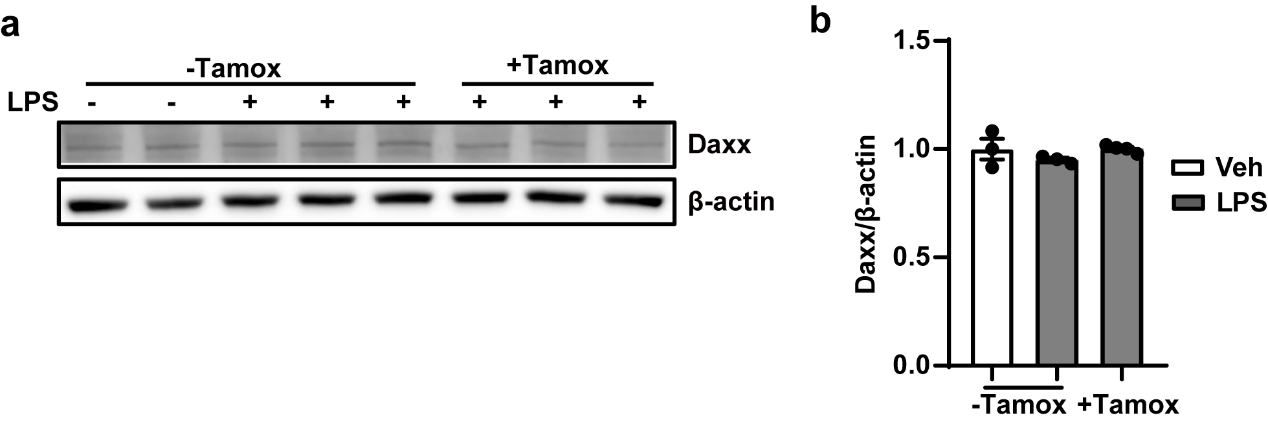


**Supplementary Figure 6. Daxx protein expression in the microglial Pdcd4 knockout mice.** (a-b) The change of protein levels of Daxx in the PFC of control or tamoxifen treatment mcKO mice after LPS administration. n=3-4 per group, one-way ANOVA and Tukey’s multiple comparisons test (F_2,7_=1.19, P=0.35).


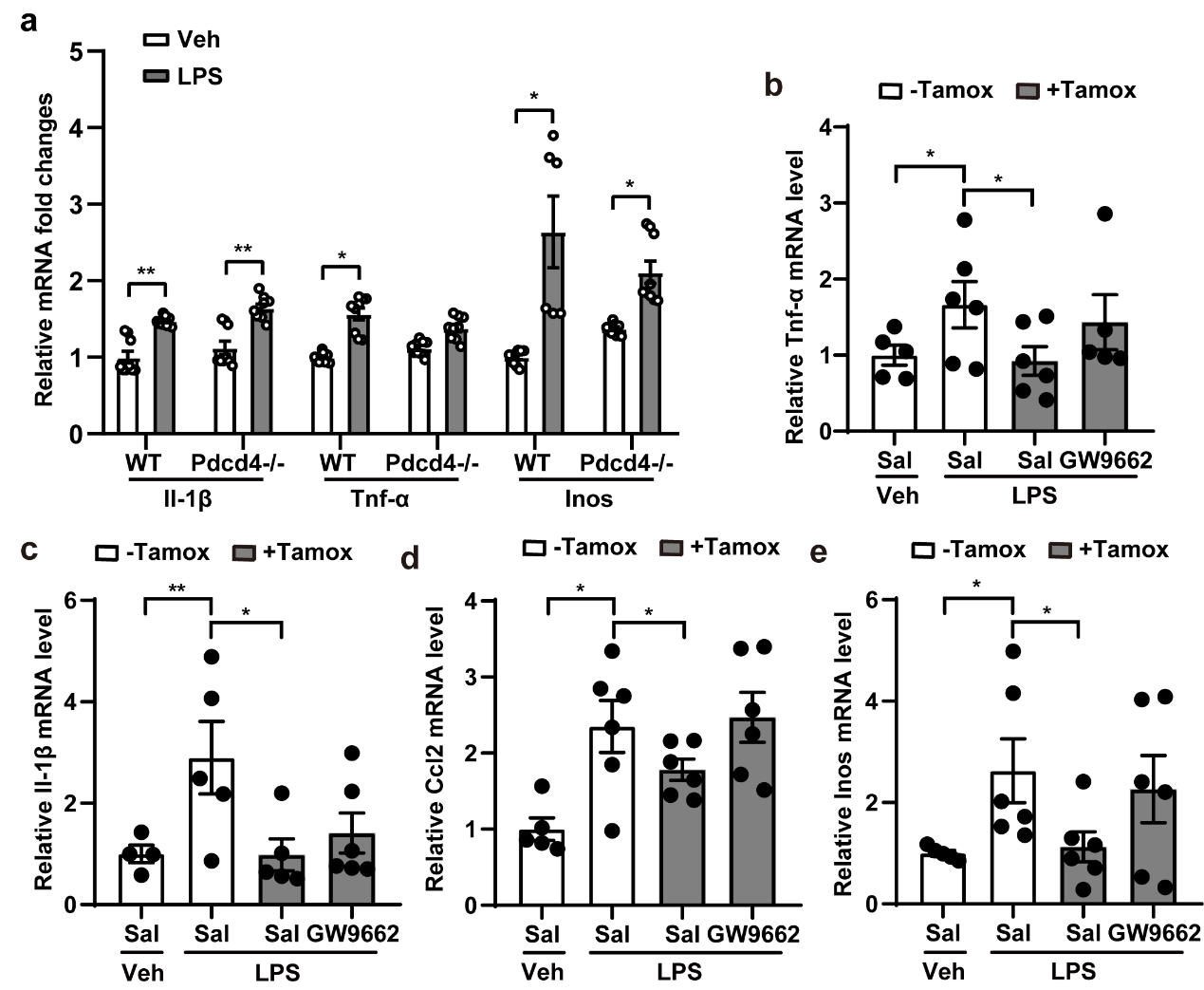


**Supplementary Figure 7. GW9662 doesn’t increase the levels of pro-inflammatory cytokines in microglial Pdcd4 knockout mice.** (a) The mRNA expression of vehicle or 1ug/ml LPS treatment for 18h in the WT or Pdcd4-/- derived microglias. N=3 per group, two-ways ANOVA and Sidak’s multiple comparisons test (Il-1β: Veh vs. LPS F_1,32_=61.34, P<0.01; WT vs. Pdcd4-/- F_1,32_=5.32, P<0.05; Tnf-α: Veh vs. LPS F_1,32_=67.96, P<0.01; WT vs. Pdcd4-/- F_1,32_=0.41, P=0.52; Inos: Veh vs. LPS F_1,29_=39.67, P<0.01; WT vs. Pdcd4-/- F_1,29_=0.16, P=0.68), *P<0.05, **P<0.01. (b-e) The change of mRNA levels of Tnf-α (F_3,18_=1.88, P=0.168), il-1β (F_3,16_=3.71, P<0.05), Ccl2 (F_3,19_=5.92, P<0.01) and iNos (F_3,19_=2.58, P=0.08) in the PFC of mcKO mice after LPS and GW9662 administration. N=5-6 per group, one-way ANOVA and Tukey’s multiple comparisons test, *P<0.05, **P<0.05.
